# Extended-spectrum beta-lactamase-producing Salmonella enteritidis in Trinidad and Tobago.

**DOI:** 10.3201/eid0501.990128

**Published:** 1999

**Authors:** B P Cherian, N Singh, W Charles, P Prabhakar


16. Gear JHS, Bevan C. An outbreak of tick-bite fever. S Afr Med J 1936;10:485-8.

### Extended-Spectrum Beta-Lactamase-Producing *Salmonella* Enteritidis in Trinidad and Tobago

**To the Editor:** *Salmonella* Enteritidis, a predominantly localized pathogen of the human gastrointestinal tract, can become invasive in very young, very old, malnourished, and immunocompromised patients. In recent years, *S.* Enteritidis has emerged as a major intestinal pathogen in Trinidad and Tobago (population 1.2 million); in 1997, *S.* Enteritidis caused 79 (66%) of 119 culture-confirmed salmonella infections, in contrast to 18 (18%) of 99, 48 (47%) of 102, and 107 (61%) of 178 in 1994, 1995, and 1996, respectively. Increased incidence of *S.* Enteritidis infections has been reported worldwide (1,2). Of 216 human *S.* Enteritidis isolates tested for antimicrobial susceptibility between 1994 and 1996 in Trinidad, none were resistant to cephalosporins, aminoglycosides, ampicillin, trimethoprim-sulphamethoxazole, chloramphenicol, and norfloxacin/ciprofloxacin by the Kirby-Bauer disk diffusion method, which uses the National Committee for Clinical Laboratory Standards (NCCLS) breakpoints (3).

Here we report an unusual isolate of *S.* Enteritidis resistant to all penicillins and cephalosporins—including third-generation cephalosporins, gentamicin, tobramicin, and trimethoprim-sulphamethoxazole—by the Kirby-Bauer disk diffusion method. Amoxicillin-clavulanate and piperacillin-tazobactam disks gave zone sizes of 15 mm and 19 mm, respectively, which are classified as intermediate in the NCCLS guidelines. This isolate was recovered from the blood culture of a febrile, nonneutropenic patient with multiple myeloma on two occasions 24 hours apart in March 1998. The isolate was sensitive only to ofloxacin and imipenem. Admitted to the hospital with compressed fracture of the spine for physiotherapy in December 1997, the patient had several febrile episodes and received several courses of multiple empirically prescribed antibiotics (cefotaxime, gentamicin, and piperacillin). The patient had not traveled abroad during the previous 6 months.

Because cephalosporin resistance in salmonellae has not been reported before in the Caribbean, we investigated the mechanism behind this third-generation cephalosporin resistance further. Using amoxicillin-clavulanate in combination with ceftazidime, ceftriaxone, and aztreonam, we performed the double disk synergy test to determine whether this strain was an extended-spectrum beta-lactamase producer as described elsewhere (3); augmentation of the zone at the junction of amoxicillin-clavulanate and aztreonam/ceftriaxone/ceftazidime zones confirmed that indeed it was.

In the past few years, third-generation cephalosporin resistance in *S.* Enteritidis has been described in Europe (4), the United States (5), Turkey (6), India (7,8), and Argentina (9). Few reports exist of extended-spectrum beta-lactamase–mediated third-generation cephalosporin resistance in *Salmonella* spp. To our knowledge, this is the first report of this type of resistance among *S.* Enteritidis in the Caribbean. This patient was treated with ciprofloxacin for 1 week; subsequent blood cultures were negative.

This unusual isolate highlights the need to establish an antimicrobial resistance surveillance network for Salmonella isolates, including *S.* Enteritidis, to monitor the trends and new types of resistance mechanisms in the Caribbean. An epidemiologic study of *S.* Enteritidis infections is being planned to describe the extent of the problem and to define risk factors and vehicles of human infections in three Caribbean countries, including Trinidad and Tobago.

**B.P. Cherian,* Nicole Singh,* W. Charles,* and P. Prabhakar*†**

*Port of Spain General Hospital, Port of Spain, Trinidad; and †Caribbean Epidemiology Center (CAREC), Port of Spain, Trinidad

### References

1. Centers for Disease Control and Prevention. Salmonella surveillance annual tabulation summary, 1993-1995. Atlanta: U.S. Department of Health and Human Services; 1997.
2. Communicable Disease Surveillance Center. Salmonella in humans: PHLS salmonella data set, England and Wales, 1981-1996. London: The Center; 1997.
3. National Committee for Clinical Laboratory Standards. Performance standards for the anti-microbial disk susceptibility tests for bacteria that grow aerobically. Approved standard M7 - A4. Villanova (PA): The Committee; 1997.

4. Fantin B, Pangon B, Potel G, Caron F, Vallee E, Vallois JM, et al. Activity of sulbactam in combination with ceftriaxone in vitro and in experimental endocarditis caused by *Escherichia coli* producing SHV-2-like beta- lactamases. Antimicrob Agents Chemother 1990:34;581-6.
5. Morosini MI, Blasquez J, Negri MC, Canton R, Loza E, Baquero F. Characterization of a nosocomial outbreak involving an epidemic plasmid encoding for TEM-27 in *Salmonella enteritidis.* J Infect Dis 1996;174:1015-20.
6. Herikstad H, Hayes PS, Hogan J, Floyd P, Snyder L, Augulo FJ. Ceftriaxone resistant Salmonella in the United States. Pediatr Infect Dis J 1997;16:904-5.
7. Vahaboglu H, Hall LM, Mulazimoglu L, Dodanli S, Yildirim I, Livermore DM. Resistance extended spectrum cephalosporins caused by PER-1 beta lactamase, in *Salmonella typhimurium* in Istanbul, Turkey. J Med Microbiol 1995;43:294-9.
8. Kumar A, Nath G, Bhatia BD, Bhargava V, Loiwal V. An outbreak of multidrug resistant *Salmonella typhimurium* in a nursery. Indian Pediatr 1995;(3)980:881-5.
9. Wattal C, Kaul V, Chigh TD, Kler N, Bhandari SK. An outbreak of multi drug resistant *Salmonella typhimurium* in Delhi (India). Indian J Med Res 1994;100:266-7.
10. Rossi A, Lopardo H, Woloj M, Picanet A, Marino M, Galds M, et al. Non-typhoid Salmonella spp. resistant to cefotaxime. J Antimicrob Chemother 1995;36:697-702.

### New *emm* (M Protein Gene) Sequences of Group A Streptococci Isolated from Malaysian Patients

**To the Editor:** We analyzed the M-type–specific *emm* gene sequences of 24 random *Streptococcus pyogenes* isolates from sterile- and nonsterile-site clinical specimens of Malaysian patients. In contrast to isolates in the United States, which rarely have new *emm* sequences, 6 of these 24 Malaysian isolates had new *emm* gene sequences, which suggests a large reservoir of group A streptococci expressing new M-type specificities in Malaysia.

The M protein is a surface-exposed principal virulence factor of group A streptococci (GAS) and a potential vaccine candidate. The hypervariable M-type–specific N-terminal portion of the M molecule extends from the cell wall and evokes protective antibodies. Approximately 75 M antigenic types of GAS are recognized, and several provisional types have been proposed (1). Formulation of a universally effective vaccine is complicated by the M-type–specific nature of protective anti-GAS antibodies, temporal and geographic variations in GAS M-type prevalence (2), and lack of information on GAS M types from areas where rheumatic fever and rheumatic heart disease, sequelae of GAS pharyngitis, are endemic (3). The lack of specific M-typing antisera is also a limiting factor in determining type distribution. Recently, Beall and colleagues (4,5) demonstrated that sequence analysis of the hypervariable portion of the *emm* gene encoding M-type specificity (*emm* typing) was an alternative when M-typing antisera were not available.

Attempts to type selected Malaysian strains of GAS by M protein status have yielded poor results. Fewer than 16% of strains were typable with standard M-typing antisera (6). The existence of new M types in Southeast Asia was suggested as an explanation. To investigate this possibility, we subjected 27 selected strains (6 from blood, 15 from pharyngitis, 3 from pus, and 3 pharyngeal carrier cultures) collected between January 1994 and December 1996 to *emm* typing. Initial isolation, serogrouping, T typing, and determination of opacity factor production were performed in Kuala Lumpur, by standard techniques, commercial media, reagents, and antisera (7). Strains were transported to the Centers for Disease Control and Prevention in Atlanta, Georgia, USA, for *emm* typing, where serogrouping, T typing, and opacity factor determinations were repeated, and *emm* typing was performed (4,5). DNA sequences were subjected to homology searches against all known *emm* sequences by Genetics Computer Group Software, Version 9. (Most sequences in this database were found in strains isolated from patients living in Europe and North America.)

Of the 27 cultures analyzed, 24 were GAS, 2 were group G streptococci, and 1 was nongroupable Streptococcus. Ten of the 24 GAS strains were standard *emm* types *emm*3, *emm*12, *emm*22, *emm*60, and *emm*76 (encoding the classic M types M3, M12, M22, M60, and M76, respectively); 4 were the provisional *emm* types *pt180*, *pt2841*, and *pt5757*; and 3 were previously identified *emm* sequence types *st64/14* and *st2034* (GenBank accession numbers X72932 and U74320, respectively). The *st2034* sequence, originally identified in children from Papua New Guinea, has also been found in Brazil, California, and Hawaii (B. Beall, R. Facklam, unpub. data). One GAS had a sequence previously found in group G streptococci (*emmLG6*, accession number U25741). Finally, 6 were of five new *emm* sequence types discovered in this study

